# Antiproliferative and Apoptosis Triggering Potential of Paclitaxel-Based Targeted-Lipid Nanoparticles with Enhanced Cellular Internalization by Transferrin Receptors—a Study in Leukemia Cells

**DOI:** 10.1186/s11671-018-2688-x

**Published:** 2018-09-06

**Authors:** Yang Dai, Jingcao Huang, Bing Xiang, Huanling Zhu, Chuan He

**Affiliations:** Department of Hematology, Hematology Research Laboratory, West China Hospital, Sichuan University, No. 37 GuoXue Xiang, Chengdu, Sichuan 610041 People’s Republic of China

**Keywords:** Leukemia, Paclitaxel, Lipid nanoparticles, Apoptosis, Cancer targeting, Transferrin

## Abstract

Leukemia is a typical blood cancer that is characterized by the numerous duplication and proliferation of white blood cells. The main aim of this study was to develop PTX-loaded multifunctional nanoparticles and target to leukemia cells. In this study, transferrin-decorated paclitaxel-loaded lipid nanoparticle (TPLN) was prepared with an aim to increase the chemotherapeutic efficacy in the leukemia cells. Results clearly showed the superior targeting potential of TPLN to the HL-60 cancer cells compared to that of the paclitaxel-loaded nanoparticles (PLN). To be specific, TPLN showed a significantly higher cytotoxic effect in the cancer cells compared to that of the PLN indicating the superior targeting efficiency of the Tf-decorated nanoparticle system. The IC50 value of TPLN was 0.45 μg/ml compared to 2.8 μg/ml for PLN. TPLN induced a most remarkable apoptosis of the cancer cells and much of the cells were distorted with huge presence of the apoptotic body formation. Importantly, TPLN showed a remarkable reduction in the viable cells proportion to ~ 65% with around ~ 30% apoptosis cells (early and late apoptosis). Overall, results clearly showed the targeting potential of ligand-conjugated lipid nanoparticle system to the leukemia cells that might pave the way for the successful cancer treatment.

## Background

Leukemia is a typical blood cancer that is characterized by the numerous duplication and proliferation of white blood cells [1]. The leukemia therefore severely affects the lymphatic system and bone marrow and results in high mortality in several patients. The abnormal hematopoietic stem cells causes uncontrolled multiplication in the bone marrow and results in the production of immature WBC [2, 3]. Chemotherapy is the most preferred choice of treatment of this disease; however, most of the chemotherapeutic drugs did not result in treatment efficacy and causes severe toxicity to the normal tissues [4, 5]. Therefore, immediate treatment of leukemia is essential for the survival of cancer patients. A new delivery strategy that could increase the efficacy of drug while reduce its toxic effect is the need of the time [6].

Paclitaxel (PTX) is regarded as one of the potent drug in the treatment of multiple cancers including leukemia [7]. The potential clinical effect of PTX is severely hampered by it systemic side effects such as myelosuppression to the healthy tissues. Moreover, free drug were reported to quickly eliminate from the systemic circulation without eliciting its therapeutic effect [8]. Therefore, it is important to design the suitable delivery system to enhance the stability and therapeutic effect of the anticancer drug. Among multiple carrier system, lipid nanoparticles are reported to possess excellent systemic stability [9, 10]. To be specific, solid lipid nanoparticles (SLN) are a unique class of attractive nanocarrier system that prevent the chemical degradation of the encapsulated drug and increases the bioavailability [11]. The salient features of SLN includes excellent stability, controlled release of drug, stable at room temperature, use of biocompatible and biodegradable lipid system, and high entrapment efficiency of lipophilic drugs such as paclitaxel. The nanoparticle encapsulation of drug is expected to accumulate in the tumor in a preferential manner owing to the enhanced permeation and retention (EPR) effect [12–14]. Although nanoparticle could increase the efficacy of encapsulated drug; however, target specificity to tumor is still a question. To address this issue, nanoparticle could be decorated with the targeting ligand which could specifically target the receptors overexpressed in cancer cells and increase the drug accumulation and efficacy [15, 16]. In this study, we have employed transferrin which will bind specifically to the transferrin receptor which is overexpressed in leukemia cells. Leukemia is known to overexpress huge numbers of transferrin receptors. The transferrin-conjugated nanoparticle will be accumulating in the cancer cells in a receptor-mediated fashion [17].

In this study, we have developed a multifunctional nanoparticle consisting of transferrin-decorated solid lipid nanoparticles encapsulated with paclitaxel. To incorporate the transferrin to the SLN, we have synthesized a T_f_-PEG-oleic acid conjugate by the conjugation of PEG and oleic acid which was then conjugated with the transferrin. The presence of PEG will increase the stability of nanocarriers in the in vitro and systemic environment. Besides, the presence of PEG on the outer surface of carrier will enhance the circulatory potential of carrier system and thereby the therapeutic efficacy.

Overall, the main aim of this study was to develop PTX-loaded multifunctional nanoparticles and target to leukemia cells. The physicochemical aspect of nanoparticle was investigated. The cytotoxic effect of free drug and drug-loaded formulation was tested in leukemia cancer cells. Cellular uptake experiment was performed to portray the target specificity of transferrin ligand. The superior anticancer effect of targeted nanoparticle was further studied by Hoechst 33342 apoptosis assay and then quantitatively studied using flow cytometer after staining with Annexin V and PI.

## Methods

### Materials

Compritol 888 ATO (Glycerol behanate) was kindly provided by Gattefosse (China). Cholesterol, oleic acid, and paclitaxel were purchased from Sigma-Aldrich, China. Human transferrin (Tf) was purchased from Sigma-Aldrich, China. All other chemicals were of reagent grade and used as such.

### Preparation of Paclitaxel-Loaded Transferrin-Conjugated Solid Lipid Nanoparticles

Transferrin-PEG-oleic acid (Tf-PEG-OA) conjugate was synthesized based on amide bond interaction [18]. Briefly, oleic acid, NHS, and DCC at a molar ratio of 1:2:2 were dissolved in organic solvent and stirred for 16 h. The reaction was carried out at room temperature. Separately, NH2-PEG-COOH was dissolved in DMSO and triethylamine was added and reaction was allowed until 12 h under inert atmosphere. The so-formed PEG-OA was collected by dialysis and freeze drying process. Now, PEG-OA, DCC, and NHS at a molar ratio of 1:2:2 were dissolved in DMSO and transferrin (along with TEA) was added to the reaction mixture and reaction was carried out in inert atmosphere for 12 h. The so-formed Tf-PEG-OA containing formulation was dialyzed for 48 h and then lyophilized and stored under further processing of experiments.

The SLN was prepared by solvent-evaporation method [19]. Briefly, PTX, Compritol 888 ATO, cholesterol, and Tf-PEG-OA were dissolved in an organic mixture consisting of ethanol/chloroform (1:5) and stirred for 30 min. The resulting organic solution was slowly added in an aqueous phase containing 1% polyvinylalcohol and immediately homogenized using T25 homogenizer (IKA, Bangalore, India) at 12,000 rpm. The solution was then probe sonicated for 3 min. The dispersion was stirred for 12 h until all the organic solvents were evaporated. The unentrapped free drug was separated from drug-loaded nanoparticles by water three times using an Amicon Ultra-4 centrifugal filter (Millipore Corporation, Bedford, USA). The entrapment efficiency (EE) and loading efficiency (LE) was determined by HPLC method. The drug-loaded nanoparticle was methanol and stirred for 30 min and diluted with equal volume of water. The amount of drug loaded was calculated by injecting in the HPLC column. The drug-loaded lipid nanoparticle was stored at 4 °C until further use.

### Physical Characterization of Nanoparticles

The particle size and distribution of different nanoparticles were evaluated by dynamic light scattering (DLS) method using Zetasizer ZS Nano Malvern Instruments, UK. The particles were diluted with distilled water and used for the analysis. The study was performed at room temperature in triplicates.

The morphology of nanoparticles was observed using transmission electron microscope (TEM) using JEM-2000EX (JEOL, Japan). The nanoparticles were stained with phosphotungistic acid and allowed to stay for 15 min. The particles were then drained off the water and dried in an infrared light for 5 min. The particles were imaged using TEM.

### In Vitro Drug Release Study

The in vitro drug release characteristics of two nanoparticles were studied using dialysis method. Briefly, 1 ml of nanoparticles dispersions were packed in a dialysis membrane bag (MWCO: 3500) with both the ends of the membrane sealed properly. The release experiment was initiated by placing the end-sealed dialysis bag in 10 ml of release medium in an automatic shaker at a speed of 100 rpm. At specific time point, 1 ml of sample was collected and analyzed for drug release using HPLC. Perkin Elmer HPLC system (Norwalk, USA) equipped with a pump (series 200), on-line vacuum degasser (series 200), auto sampler (series 200), column oven (series 200), a UV/VIS detector (series 200) was used. C18 column (250 mm × 4.6 mm, 5 μm) protected with pre-column guard cartridge RP18 (30 × 4.6 mm, 10 μm; Norwalk, USA) was used. A linear gradient was applied; 0 min, 10% acetonitrile; 30 min, 90% acetonitrile and analyzed at 214 nm.

### Cytotoxicity Assay

The cytotoxicity of free drug and drug-loaded nanoparticles were evaluated by 4,5-(dimethylthiazol-2-yl) 2,5-diphenyl-tetrazolium bromide (MTT) assay. The HL-60 cells at a seeding density of 8000 cells per well were seeded in a 96-well plate and incubated for 24 h. The cells were then treated with free drug and drug-loaded formulations at different concentrations and further incubated for 24 h at 37 °C in humidified CO_2_ (5%) incubator. The cells were then treated with 10 μl of MTT solution (5 mg/ml) for 4 h. The cells were then exposed to DMSO and incubated for 20 min. The formazan crystals were then studied for absorbance using a microplate reader (Multiskan GO, USA) at 570 nm. The amount of formazan absorbance is directly proportional to the amount of viable cells present in each well.

### Cellular Uptake of Nanoparticles

The targeting efficiency of nanoparticles toward the leukemia cells were evaluated by the cellular uptake experiment at the in vitro level. Briefly, HL-60 cells were seeded in a 6-well plate at a seeding density of 3 × 10^5^ cells per well. The cells were incubated for 24 h prior to the treatment. In order to observe the fluorescence and nanoparticle tracking, nanoparticle was loaded rhodamine B as a fluorescent dye. The dye-loaded nanoparticle was exposed to the cancer cells and incubated for 3 h. The cells were then washed and mounted on a glass slide. The cells were then observed using a confocal laser scanning microscope (CLSM, Nikon, Japan).

### Hoechst 33342 Assay

The qualitative apoptosis assay was performed using Hoechst 33342 staining. Briefly, HL-60 cells were seeded in a 6-well plate at a seeding density of 3 × 10^5^ cells per well. The cells were incubated for 24 h prior to the treatment. The cells were treated with respective formulations and incubated for 24 h. The cells were then washed two times with PBS and fixed with 4% paraformaldehyde solution for 10 min. The apoptosis in cells were then observed using a fluorescent microscope.

### Flow Cytometer-Based Apoptosis Assay

The quantitative apoptosis was evaluated by flow cytometer by staining with Annexin V and PI. Briefly, HL-60 cells were seeded in a 6-well plate at a seeding density of 3 × 10^5^ cells per well. The cells were incubated for 24 h prior to the treatment. The cells were then treated with free drug and drug-loaded formulations at different concentrations and further incubated for 24 h at 37 °C in humidified CO2 (5%) incubator. The next day, cells were washed properly and cells were extracted carefully. The cells were centrifuged and pellet was treated with 2.5 μl of Annexin V and 2.5 μl of PI, respectively and incubated for 15 min. The volume was then made up to 1 ml and cell sorting was performed using a flow cytometer (BD FACS, Biosciences, USA).

### Colony Formation Assay

The 1000 cells/well were seeded in a 6-well plate with volume of media adjusted to 2 ml. The cells were allowed to form a colony for 2 days. The cells were treated with different formulations with a dose of PTX equivalent to 0.1 μg/ml and incubated for 10 days. The plates are washed with PBS and fixed in methanol and stained with hematoxylin and washed and air dried. The colony density was counted using MultimageTM light cabinet (Alpha Innotech Corporation, San Leandro, CA) with the help of AlphaEaseFCTM software.

### Statistical Analysis

The significance of differences was assessed using two-tailed Student’s *t* tests or one-way ANOVA. Differences were considered to be significant at a level of *p* < 0.05.

## Results and Discussion

**Fig. 1:**
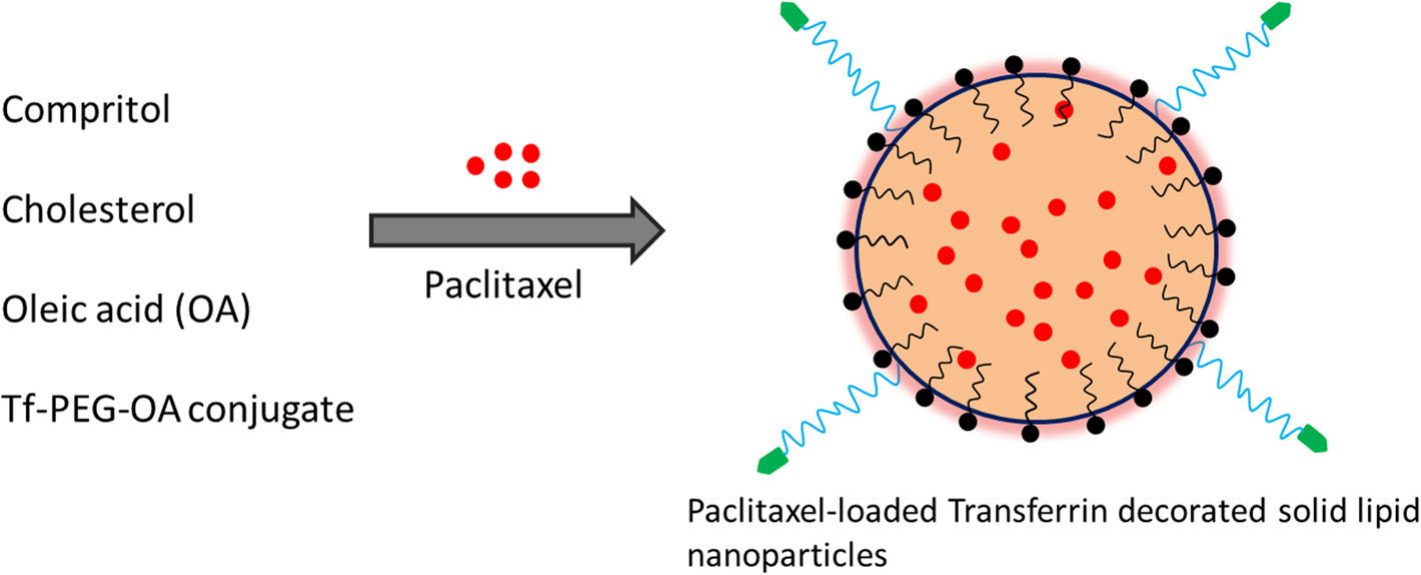
Schematic presentation of preparation of PTX-loaded transferrin-decorated solid lipid nanoparticles

Leukemia is a typical blood cancer that is characterized by the numerous duplication and proliferation of white blood cells. In this study, we have developed a multifunctional nanoparticle consisting of transferrin-decorated solid lipid nanoparticles encapsulated with paclitaxel. To incorporate the transferrin to the SLN, we have synthesized a Tf-PEG-oleic acid conjugate by the conjugation of PEG and oleic acid which was then conjugated with the transferrin (Fig. 1). The PEG-oleic acid-Tf is used for multiple purposes in the particles preparation. The presence of Tf ligand on the particle surface will enhance the target specificity of particles toward the receptor-overexpressed cancer cells. The presence of PEG on the outer surface will provide the much needed steric balance that will confer the stability to the individual particles. We have added this note to the manuscript.

### Physicochemical Characterization of Nanoparticles

**Fig. 2:**
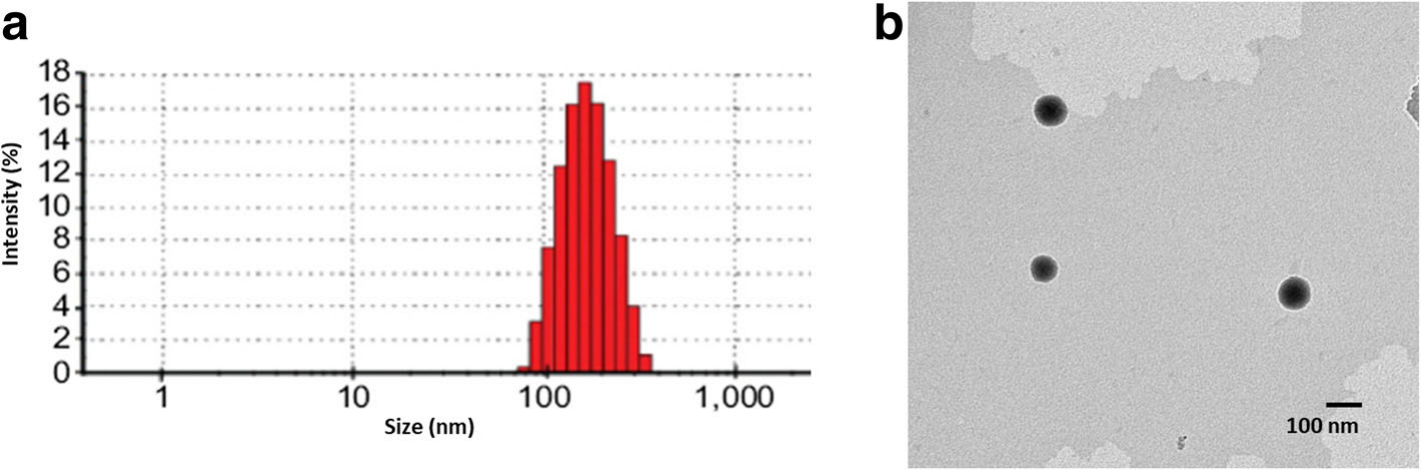
**a** Particle size distribution of TPLN measured by dynamic light scattering (DLS) analysis. **b** Particle shape measurement by transmission electron microscope (TEM). The DLS experiments were performed in triplicate (*n* = 3)

The drug-loaded nanoparticle was prepared by solvent-evaporation method followed by homogenization. The average particle size of PTX-loaded lipid nanoparticles (PLN) was ~ 140 nm while the final particle size of transferrin-decorated PTX-loaded lipid nanoparticle (TPLN) was ~ 160 nm (Fig. 2a). The slight increase in particle size was attributed to the conjugation of transferrin to the nanoparticle surface. It has been reported that particle characteristics include size and charge are important factors for its systemic performance. The size and charge plays an important role in the cellular uptake and toxic effect to the cells. A particle size of less than 200 nm as observed in the present study is reported to be most favorable for the cancer targeting as it will allow the specific accumulation of the particles in the cancer tissue owing to the enhanced permeation and retention (EPR) effect [9, 20, 21]. Similarly, surface charge of nanoparticle determines the colloidal stability particle interaction with the cell surface. The mean surface charge of TPLN was − 22.5 ± 1.56 mV indicating its potential for cancer targeting. The morphology of the particle was in turn studied by TEM (Fig. 2b). The particles were perfectly spherical in shape and uniformly distributed in the TEM grid. The uniform distribution of the particles indicates the success of the preparation methodology.

### Drug Loading and In Vitro Drug Release

**Fig. 3:**
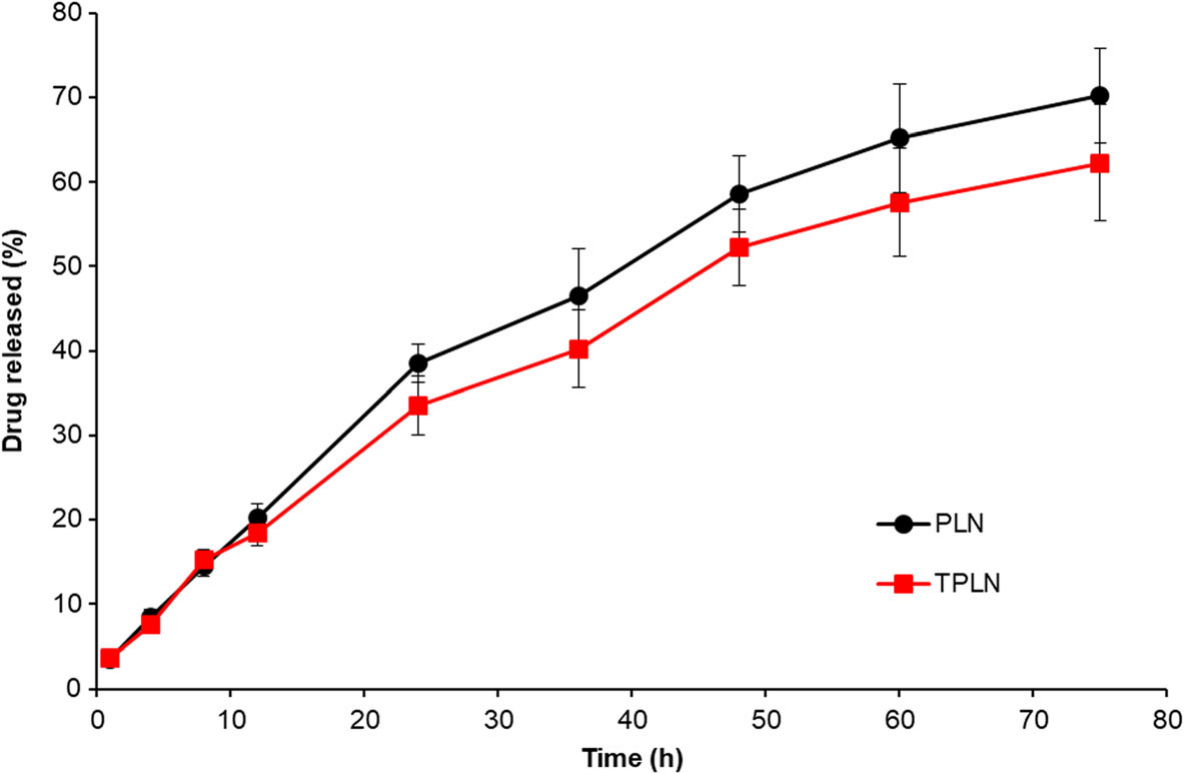
In vitro drug release characteristics of PTX from PLN and TPLN from phosphate buffered saline (pH 7.4). The drug release was studied by dialysis method and the drug is quantified by HPLC method. The measurement was performed *n* = 3

The entrapment efficiency of PTX in nanoparticle was observed to be 92.5 ± 1.35% with an active loading efficiency of 8.6% *w*/*w*. The in vitro drug release characteristics of PLN and TPLN were studied by dialysis method. The PLN and TPLN showed a sustained release of drug from the carrier system indicating its suitability for cancer targeting applications. For example, ~ 30% of drug released from both the nanoparticles at the end of 24 h study period (Fig. 3). Similarly, drug release was sustained until the end of 75 h with an average release around ~ 65% was observed. It should be noted that TPLN showed far more sustained release of drugs compared to PLN. Slightly lower release of drug was mainly attributed to the conjugation of transferrin ligand to the surface of the nanoparticle. The high molecular weight of transferrin might inhibit the release of drug to a great extent. Overall, sustained and prolonged release of drug from the nanoparticle indicates that drug is dispersed well with the lipid matrix which avoided the initial burst release or biphasic release pattern. Overall, a controlled release of drug from the carrier system might potentiate the cancer treatment.

### Cellular Uptake of the Nanoparticles

**Fig. 4:**
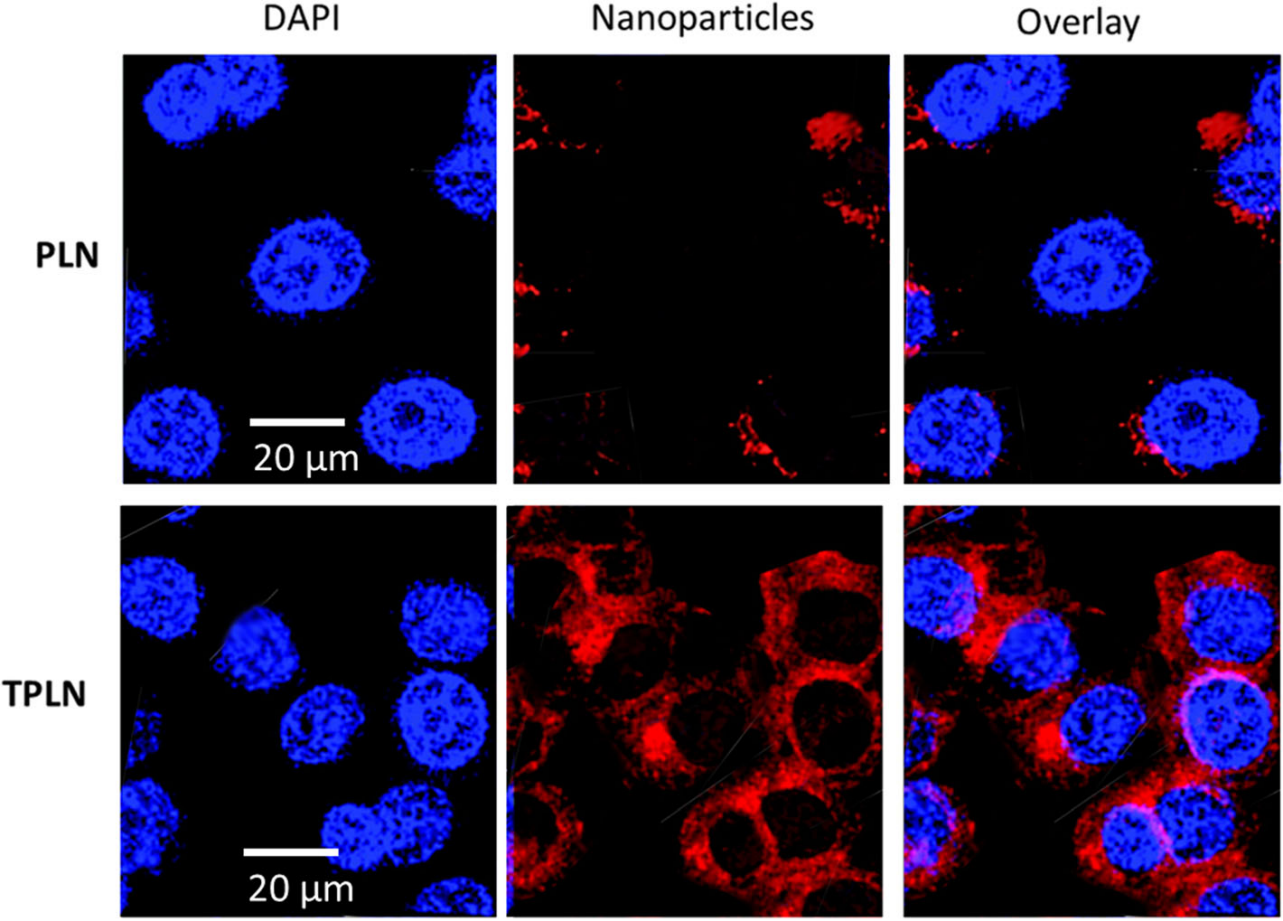
Cellular uptake analysis of TPLN and PLN using confocal laser scanning microscope (CLSM) at 37 °C. Rhodamine B was used as a fluorescent dye and incubated for 3 h. The cellular uptake analysis was performed after incubating the nanoparticles for 3 h under 37 °C in incubator. Scale bar is 20 μm

The targeting potential of the nanoparticles toward the leukemia cancer cell was tested by confocal laser scanning microscopy (CLSM). The nanoparticles were exposed to the cancer cells and incubated for 3 h. Rhodamine B was used as a fluorescent dye to track the nanoparticle distribution in the cancer cells. As shown in Fig. 4, red fluorescent intensity was relatively less in PLN compared to that in the TPLN. Results clearly showed the remarkably higher fluorescence in the cell cytoplasm compared to that of the PLN indicating the superior targeting potential of TPLN to the cancer cells. A remarkably higher fluorescent intensity was mainly attributed to the conjugation of transferrin to the nanoparticle surface. The Tf preferably bound to the Tf receptors overexpressed in the cancer cells and resulted in higher accumulation of the nanoparticles in the cancer cells [22].

### In Vitro Cytotoxicity Assay

**Fig. 5:**
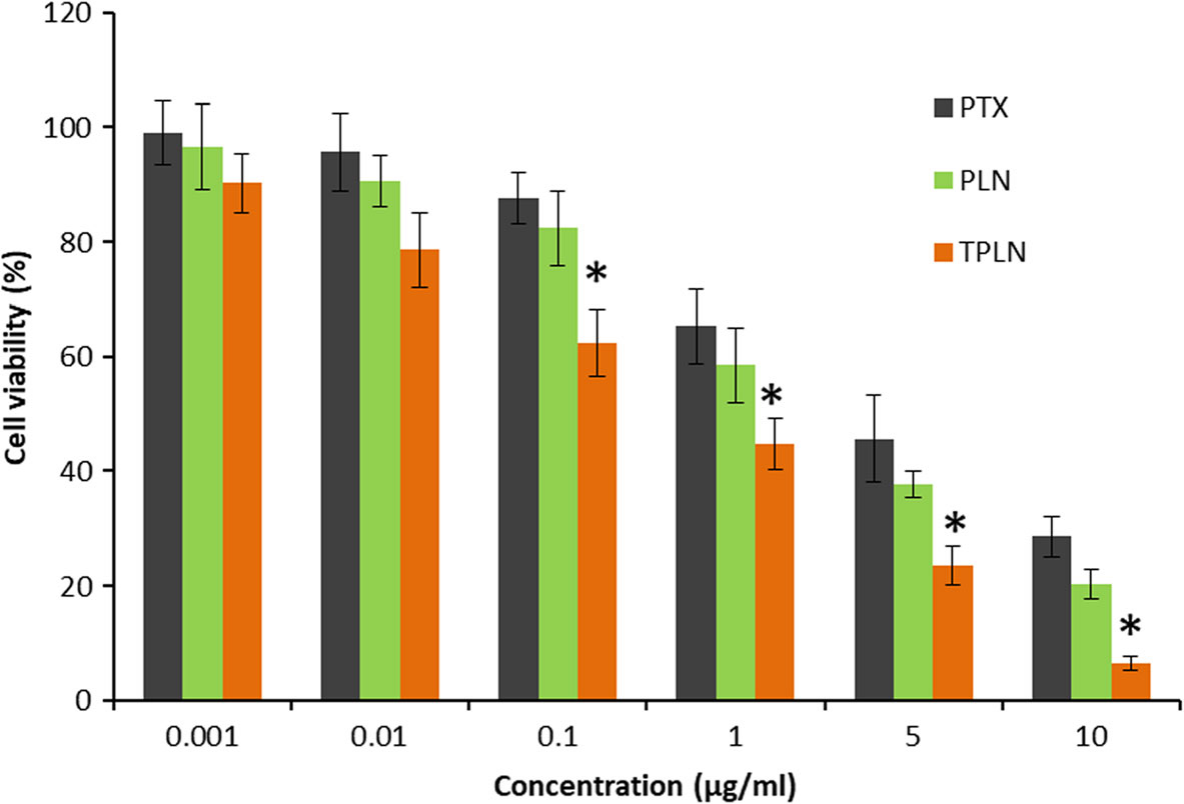
Cytotoxicity analysis of free drug and drug-loaded nanoparticle by MTT assay. After treating the cells with different concentration of drugs and incubated for 24 h, cells were analyzed by MTT assay and using a plate reader. The measurement was performed *n* = 6. *p < 0.05 is the statistical difference between TPLN and PTX

The in vitro cytotoxic effect of free PTX, PLN, and TPLN in HL-60 cells was evaluated by MTT assay. As shown in Fig. 5, all the formulations exhibited a typical dose-dependent cytotoxic effect in the leukemia cancer cells. It can be seen that although PTX showed a dose-dependent effect, however, PLN showed relatively higher cytotoxic effect which might be due to the higher intracellular uptake and sustained release of drug from the nanoparticles. To be specific, TPLN showed a significantly higher cytotoxic effect in the cancer cells compared to that of the PLN indicating the superior targeting efficiency of the Tf-decorated nanoparticle system. The IC50 value of TPLN was 0.45 μg/ml compared to 2.8 μg/ml for PLN. A sixfold decrease in the IC50 value was mainly attributed to the higher intracellular uptake of TPLN in the Tf receptor-overexpressed cancer cells. A higher delivery potential of TPLN compared to PLN and sustained release of drug to the nuclear target might be the main contributory reason for the higher cytotoxic effect in the cancer cells. Tf-decorated nanocarriers have been explored as a target to deliver anticancer drugs into cancer cells, thereby decreasing the effective concentration of administered drug and overcome the multidrug resistance (MDR)-related factors [23].

### Hoechst 33342 Apoptosis Assay

**Fig. 6:**
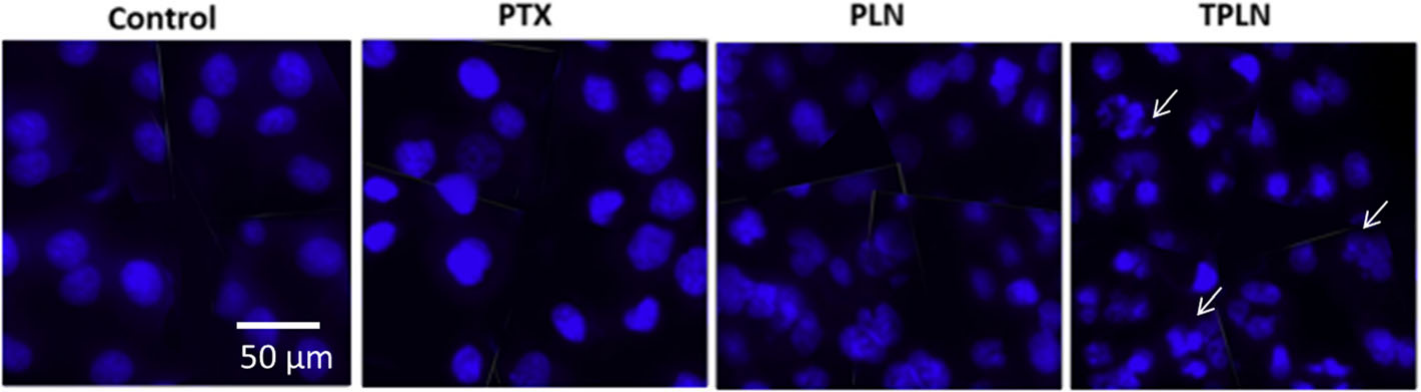
Hoechst 33342-based apoptosis assay. The cells were stained with Hoechst 33342 and the apoptosis was analyzed by florescence microscope. The cell were incubated with respective formulations for 24 h. Scale bar is 50 μm

The cytotoxic potential of nanoparticles was further evaluated by Hoechst 33342 apoptosis assay. The cells after treatment with the respective formulation was stained with Hoechst 33342 and incubated for 15 min. As seen from Fig. 6, untreated control cells were spherical with typical features for healthy cells. The PTX-treated cells however showed irregularity in the cell shape. A typical cell damage and apoptosis could be seen in the PLN-treated group that might be due to the increase uptake in the cancer cells. TPLN induced a most remarkable apoptosis of the cancer cells and much of the cells were distorted with huge presence of the apoptotic body formation. The remarkable cell cleavage and apoptosis induction was consistent with the cell viability patents as described in the preceding paragraph.

### Flow Cytometer-Based Apoptosis Assay

**Fig. 7:**
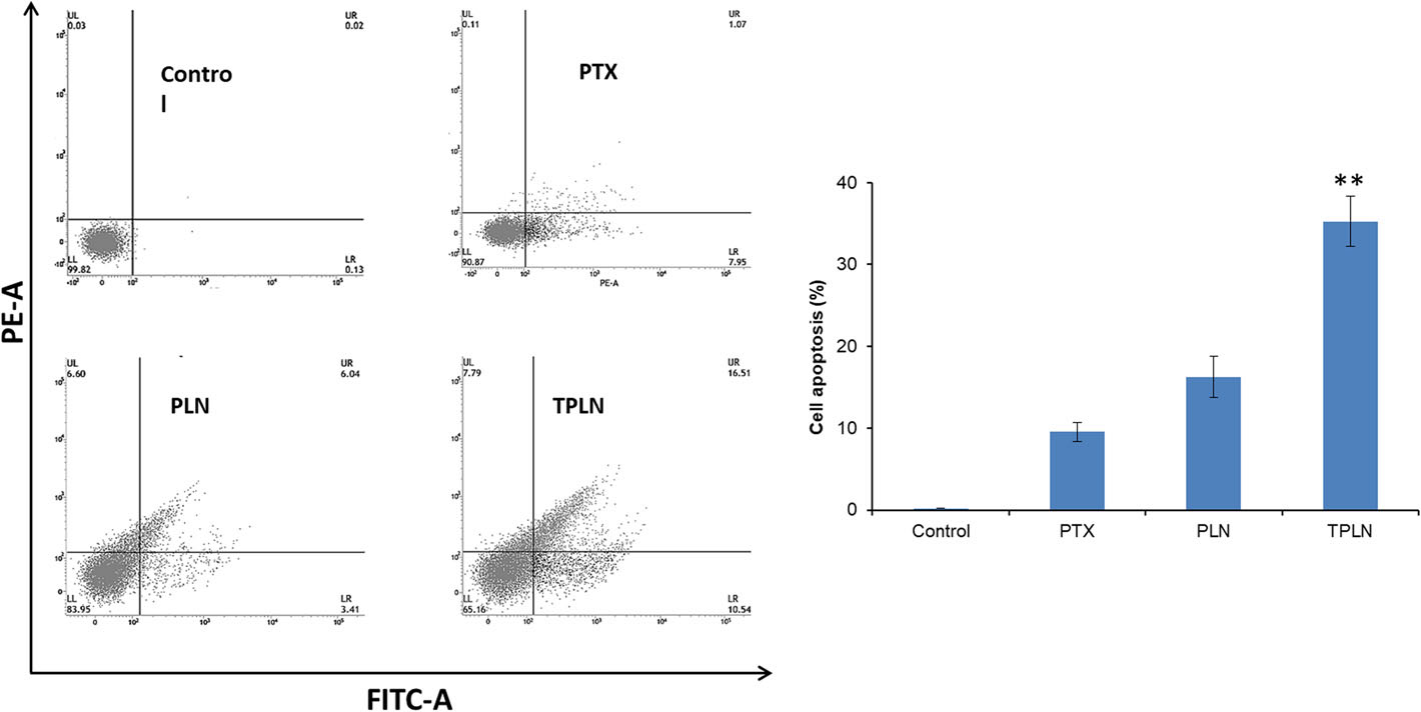
Flow cytometer-based apoptosis assay. The cells were treated with respective formulation and after 24 h, cells were stained with Annexin V and PI for 15. The cells were then sorted using flow cytometer. The cells were incubated with respective formulations for 24 h. The measurement was performed *n* = 3. *p < 0.05 is the statistical difference between TPLN and PTX

The quantitative analysis of apoptosis assay was performed by flow cytometer after staining with Annexin V and PI staining kit. The cells were treated with respective formulations and incubated for 24 h. The cells were then stained with the Annexin V and PI. As shown in Fig. 7, untreated control cells were viable with more than 99% of cells present in the viable chamber. The PTX treatment reduced the proportion of viable cells to 90% with around ~ 9% of the apoptosis. The PLN showed around ~ 10% of apoptosis cells with ~ 7% of necrotic cells. Importantly, TPLN showed a significant (*p* < 0.05) reduction in the viable cells proportion to ~ 65% with around ~ 30% apoptosis cells (early and late apoptosis). The remarkable apoptosis potential of TPLN was attributed to the receptor-mediated cellular uptake and higher accumulation of anticancer drug in the cancer cells that might enhance the anticancer effect in the tumor cells [24, 25].

### Colony Formation Assay

**Fig. 8:**
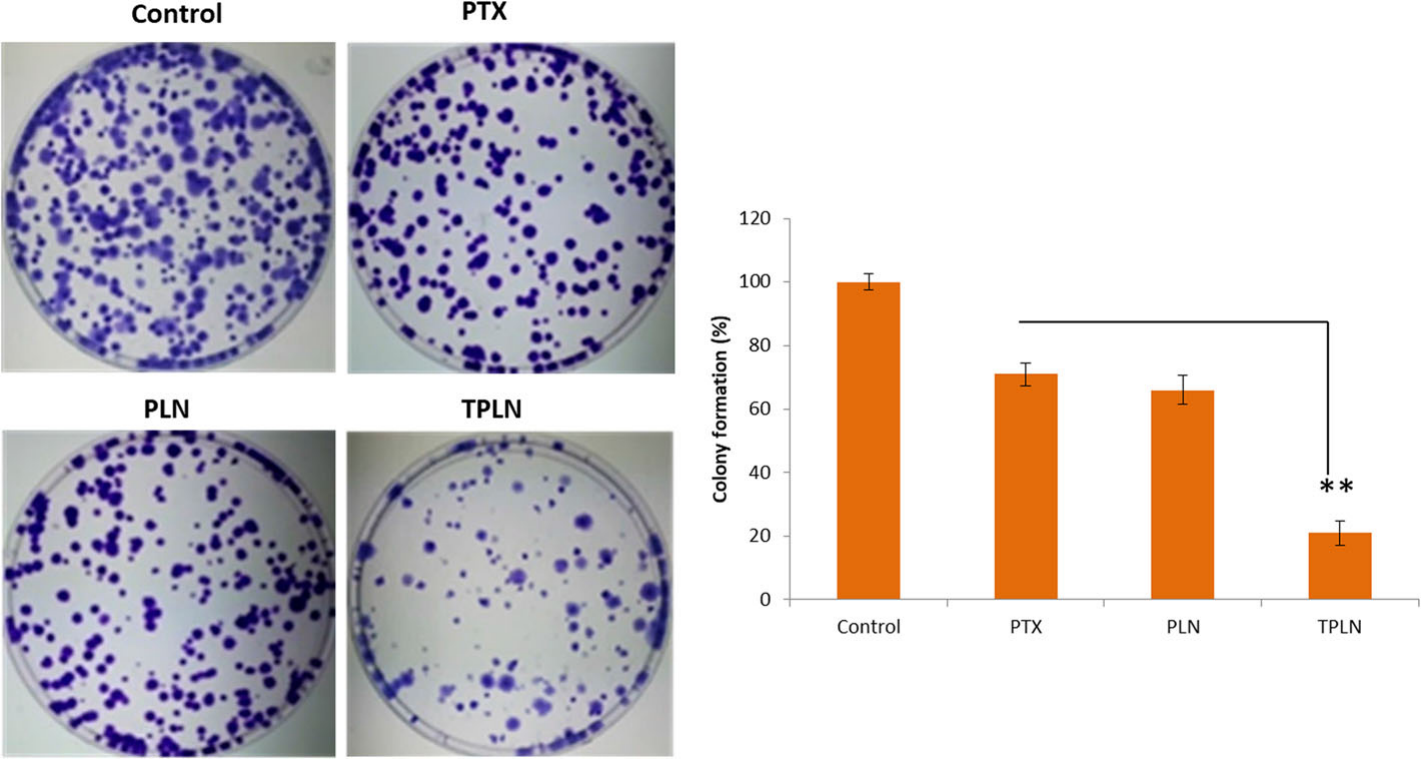
Colony formation assay. The cells were subjected to colony formation assay protocol after respective treatment. The cells were incubated with respective formulations for 24 h. **p < 0.001 is the statistical difference between PTX and PTLN

The colony formation ability in the presence of respective formulation was tested by colony formation assay Fig. 8. As shown, PTX and PLN possess slight potential to inhibit the colony formation ability of cancer cells. Importantly, TPLN showed remarkable ability to control the colony formation ability of the cancer cells. TPLN showed ~ 20% colony formation compared to ~ 70% colony formation for PTX indicating the superior anticancer effect of TPLN. The maintenance of acute myeloid leukemia is dependent on a smaller population of leukemic stem cells and progenitor cells that have the ability to form colonies; it is important to test whether TPLN is able to target the pool of colony forming cells. As the results show, it can significantly reduce the risk of leukemia cancer, suggesting an ability to prevent the development of cancer and/or eliminate early-stage malignant cells. Therefore, colony formation assay gives crucial information about the potential anticancer efficacy of the formulations.

## Conclusion

Overall, transferrin-decorated paclitaxel-loaded lipid nanoparticle was prepared with an aim to increase the chemotherapeutic efficacy in the leukemia cells. Results clearly showed the superior targeting potential of TPLN to the cancer cells compared to that of the PLN. To be specific, TPLN showed a significantly higher cytotoxic effect in the cancer cells compared to that of the PLN indicating the superior targeting efficiency of the Tf-decorated nanoparticle system. Importantly, TPLN showed a remarkable apoptosis of cancer cells and displayed strong anti-tumor ability on both HL-60 cells. Overall, results clearly showed the targeting potential of ligand-conjugated lipid nanoparticle system to the leukemia cells that might pave the way for the successful cancer treatment. The therapeutic efficacy of formulations in clinical animal model and its biodistribution will be part of our next work.

## Abbreviations

EPR: Enhanced permeation and retention effect
OA: Oleic acid
PEG: Polyethylene glycol
PLN: PTX-loaded lipid nanoparticles
PTX: Paclitaxel
SLN: Solid lipid nanoparticles
Tf: Transferrin
TPLN: Tf-conjugated PLN
